# Treatment of Subcorneal Pustular Dermatosis without Dapsone: A Case Report and Review of the Literature

**DOI:** 10.1155/2024/8140483

**Published:** 2024-04-02

**Authors:** Lindsey J. Wanberg, Brittney Schultz, Amrita Goyal

**Affiliations:** ^1^University of Minnesota Medical School, 420 Delaware St SE, Minneapolis, MN 55455, USA; ^2^University of Minnesota, Department of Dermatology, 516 Delaware Street SE, Minneapolis, MN 55455, USA

## Abstract

Subcorneal pustular dermatosis (SPD) is a rare neutrophilic dermatosis characterized by pustules on the trunk and intertriginous areas. While oral dapsone is the first-line treatment for SPD, alternative options are necessary for patients with glucose-6-phosphate dehydrogenase deficiency, drug hypersensitivity reactions, or refractory disease. To date, no consensus exists regarding next-best agents for SPD. In this report, we present a patient with significant SPD who developed dapsone hypersensitivity syndrome and then was successfully treated with colchicine and adalimumab. We propose that colchicine should be considered as a second-line treatment for SPD and present a therapeutic algorithm for clinicians to utilize when patients are not candidates for dapsone, have side effects requiring drug discontinuation, or have refractory disease.

## 1. Introduction

Subcorneal pustular dermatosis (SPD) is a rare neutrophilic dermatosis characterized by pustules on the trunk and intertriginous areas [1, 2]. It is most common in middle-aged women and may be associated with underlying systemic disorders such as rheumatoid arthritis and monoclonal gammopathy [3]. SPD presents similarly to IgA pemphigus clinically and histopathologically but can be differentiated by negative direct immunofluorescence studies [3].

Dapsone is the established first-line treatment for SPD but may not be appropriate for all patients due to refractory disease or serious potential side effects such as hemolytic anemia, agranulocytosis, or dapsone hypersensitivity syndrome (DHS) [2, 4]. To date, no consensus exists regarding next-best agents for SPD. In this report, we present a patient with SPD who developed an acute drug reaction from dapsone and then was successfully treated with colchicine and adalimumab. We review the case report literature to summarize successful treatments of SPD and propose a novel treatment algorithm with second-line and third-line treatments to consider for SPD when dapsone fails or is not tolerated.

## 2. Case

A 67-year-old female with no pertinent past medical history presented to a university dermatology clinic in December 2021 with four years of a tender and pruritic rash on her legs, trunk, breasts, and arms. The rash was refractory to clobetasol 0.05% cream, betamethasone diproprionate 0.05% cream, intralesional triamcinolone acetonide-10, and halobetasol 0.05% cream.

**Figure 1:**
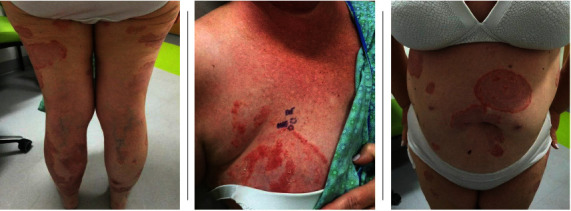
Skin findings at presentation.

Physical exam in December 2021 revealed multiple annular pink plaques studded with occasional pustules on the trunk and upper and lower extremities (see Figure 1). Repeat punch biopsies of the skin of the right breast and right thigh revealed subcorneal pustules filled with neutrophils and negative direct immunofluorescence studies. This was consistent with a diagnosis of subcorneal pustular dermatosis (also known by the eponym Sneddon–Wilkinson syndrome). Serum protein electrophoresis and serum immunofixation did not show evidence of a monoclonal gammopathy. Rheumatologic studies including ANA and rheumatoid factor were negative.

### 2.1. Treatment Course

The patient was started on dapsone 50 mg daily, up-titrating after ten days to 100 mg daily with topical corticosteroids. Glucose-6-phosphate dehydrogenase enzyme activity was within normal limits. At her one-month follow-up, her lesions showed dramatic improvement with absence of pustules and interim resolution of the patches on the abdomen.

Two days later, the patient noticed a new, pruritic erythematous macular eruption on her thighs and arms, fever with chills, but denied any facial swelling. She self-discontinued the dapsone. Laboratory studies performed three days later showed elevated liver function tests from the baseline including an aspartate aminotransferase of 191 U/L, alanine transaminase of 262 U/L, total bilirubin of 1.4 mg/dL, and alkaline phosphatase of 246 U/L. Her hemoglobin was 8.5 g/dL, and white blood cell and eosinophil count were within normal limits (7.0 × 10^9^/L and 0.4 × 10^9^/L, respectively). Given fever, transaminitis, and morbilliform eruption, there was clinical concern for dapsone hypersensitivity syndrome (DHS), a variant of drug reaction with eosinophilia and systemic symptoms (DRESS syndrome) [5]. Oral prednisone 40 mg daily was initiated, followed by a 10-week taper. Liver function tests showed improvement with this course, and the new eruption cleared within 2 weeks of drug discontinuation.

Unfortunately, around one month after dapsone discontinuation and while still on 30 mg of oral prednisone daily, the patient's SPD rash returned on the upper and lower extremities. Phototherapy with narrow-band ultraviolet-B light was denied by her insurance, so two weeks after the recurrence of rash, she was placed on acitretin 10 mg for 30 days. The acitretin helped the pruritus but failed to demonstrate improvement of skin lesions even with up-titration to 20 mg daily for an additional 60 days. Subsequently, adalimumab initiated at 40 mg every two weeks with improvement in pruritus but without change in skin lesions.

**Figure 2:**
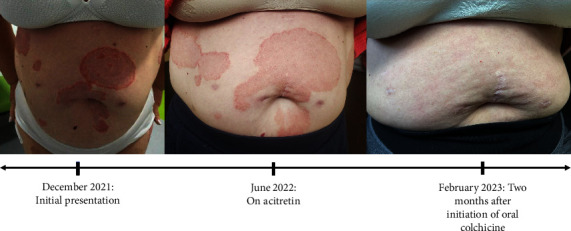
Improvement of SPD rash.

After 3 months, oral colchicine 0.6 mg daily was added. Within three weeks of starting colchicine, the patient experienced rapid significant improvement of her SPD rash and noted almost complete clearance of her trunk and upper extremity lesions; the lesions on her legs resolved within 6 weeks (see Figure 2). Because the patient had experienced an increase in mild upper respiratory infections while on adalimumab, approximately three months after starting colchicine, she attempted discontinuation of adalimumab with concomitant increase of colchicine to 1.2 mg daily, but within two weeks of her last dose of adalimumab, she began to experience recrudescence of the lesions on the legs. As a result, she resumed every-other week adalimumab injections and continued colchicine 1.2 mg daily, with significant improvement.

## 3. Discussion

This is a case of a 67-year-old female with SPD who, despite excellent disease resolution on dapsone, required discontinuation of the medication due to an acute drug reaction. Although dapsone is the established first-line treatment for SPD, it carries serious potential side effects such as hemolytic anemia, agranulocytosis, or dapsone hypersensitivity syndrome (DHS) [2, 4]. No consensus exists regarding next-best agents for SPD.

### 3.1. Colchicine for SPD

Colchicine is a low-risk medication that may be considered in neutrophilic dermatoses. It is an antineutrophilic drug that has demonstrated efficacy in neutrophilic dermatoses such as Sweet's syndrome and palmoplantar pustulosis [6]. It is relatively affordable and well tolerated, with the most common side effects being diarrhea and vomiting; however, these side effects are seen less frequently at lower doses [6]. Additionally, colchicine is considered safe to use long-term based on studies that examine its use in gout and cardiovascular disease [7].

To our knowledge, data on the efficacy of colchicine for SPD have not been summarized. We performed literature review to identify published case reports of SPD treated with colchicine. The results of our findings are summarized in Table 1. Four case reports describe colchicine leading to the resolution of symptoms, [8–11] whereas 13 case reports report no improvement on the drug. Even so, due to colchicine's low risk and ability to completely clear skin lesions in some patients, it presents significant promise as a dapsone-alternative therapy for a subset of patients with SPD. Insufficient data were included in the case reports to draw conclusions about what patient characteristics may be associated with positive response to colchicine.

### 3.2. Proposing a Treatment Algorithm

**Figure 3:**
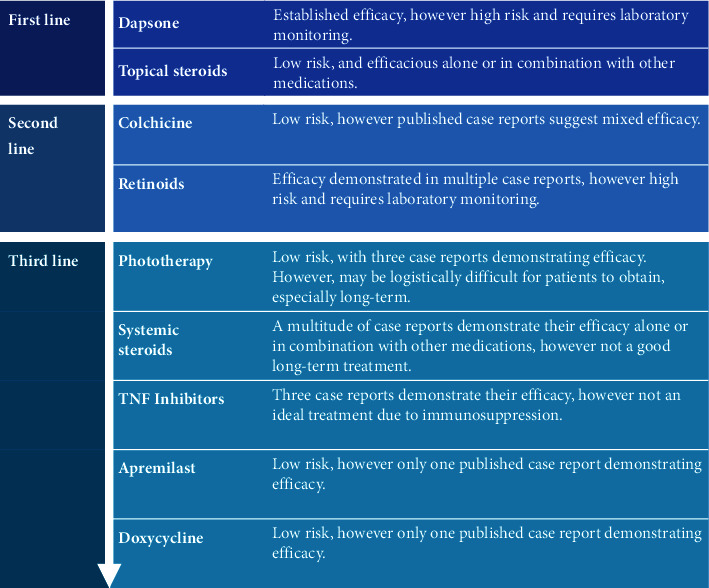
Subcorneal pustular dermatosis therapeutic algorithm.

Due to dapsone's side effect profile, we argue for early consideration of dapsone-alternatives such as colchicine in patients at risk for dapsone intolerance. This includes patients with pre-existing anemia, G6PD deficiency, or sulfa allergy or sensitivity [4]. A multitude of case reports published in the last 25 years describe success with oral retinoids, small-molecule inhibitors, phototherapy, biologics, and various topicals (Table 2). These case reports were gathered with a Boolean search on PubMed using the phrase “(“subcorneal pustular dermatosis” OR “Sneddon-Wilkinson disease”) AND (“case” OR “treatment”).” Case reports were not included in the table if they were not in English, were published before 1998, included dapsone as the primary successful treatment, and/or did not describe a successful treatment. From review of these data, we propose a novel treatment algorithm for SPD (Figure 3).

Overall, there is significant work to be done in determining safe and efficacious treatments for SPD. Our algorithm is based on case reports which are subject to publication bias and overinterpretation [56]. Further, our case describes a patient on colchicine and adalimumab simultaneously, without evaluating colchicine alone. However, given the rarity of this condition, it is unlikely that randomized controlled trials of treatments for SPD using existing agents such as dapsone, colchicine, oral retinoids, and TNF-alpha inhibitors are on the horizon. Thus, this algorithm provides a reasonable starting point for shared clinical decision-making with patients.

## Figures and Tables

**Table 1:** Colchicine therapy for subcorneal pustular dermatosis.

Case	Patient	Colchicine therapy course	Was colchicine successful?
Lao-Ang et al. Unknown date [8]	49-year-old female	Colchicine 0.5 mg/day: complete resolution after 6 weeks and no relapse at 6-month follow-up	Yes
Pavithran [9]	40-year-old male	Colchicine 0.5 mg BID and then maintenance dose of 0.5 mg/day: complete resolution after one week. No relapses at maintenance dose	Yes
Present case	67-year-old female	Colchicine 0.6 mg per day, adalimumab 40 mg every two weeks, triamcinolone 0.1% cream or augmented betamethasone 0.05% ointment twice daily as needed: improvement within 3 weeks with one flare which resolved with topical steroids. There was no improvement with adalimumab and topicals alone	Yes
Bedi [10]	28-year-old female	Colchicine 0.6 mg BID: initial response but flare at 3 months	Yes, but recurrence reported
Teraki and Sugai [11]	72-year-old female with mild IgA elevation	Colchicine, unknown dose: initial complete response but recurrence 1-2 months later	Yes, but recurrence reported
Stefanaki et al. [12]	57-year-old male with palmoplantar pustular psoriasis (PPP)	Colchicine 0.5–1.5 mg/day: started when patient had PPP eruption, not SPD (although had a recent history of SPD previously controlled with dapsone). At 12 month follow-up after starting colchicine and discontinuing dapsone, patient did not have relapse of PPP or SPD	Unclear, may have prevented recurrence
Orton and George [13]	40-year-old female	Colchicine 1 mg/day: no maintained response	No
Bonifati et al. [14]	54-year-old female	Colchicine, unknown dose: only partial control	No
Ratnarathorn and Newman [15]	45-year-old male with nodal marginal zone lymphoma	Colchicine 0.6 mg TID and fluocinonide ointment: worsening of SPD over the next 3 months. Colchicine was discontinued	No
Khachemoune and Blyumin [3]	28-year-old male	Colchicine 0.6 mg/day with dapsone 50 mg/day for 3 months: no response and had side effects such as diarrhea and weight loss	No
Berk et al. [16]	51-year-old male	Colchicine, unknown dose: no benefit	No
Berk et al. [16]	61-year-old male	Colchicine, unknown dose: no benefit	No
Voigtländer et al. [17]	79-year-old female	Colchicine, unknown dose: no benefit	No
Romagnuolo et al. [18]	80-year-old female	Colchicine, unknown dose: unknown benefit, discontinued due to “severe” gastrointestinal side effects	No
Naretto et al. [19]	37-year-old female with systemic lupus erythematosus	Colchicine, unknown dose: no benefit	No
Todd et al. [20]	71-year-old male with monoclonal IgA gammopathy	Colchicine, unknown dose: no benefit	No
Brown et al. [21]	78-year-old female with chronic lymphocytic leukemia	Colchicine 1.5 mg/day: no benefit	No
Guerin et al. [1]	69-year-old female with monoclonal IgA gammopathy	Colchicine, unknown dose: no benefit	No
Ahmad and Ramsay [22]	57-year-old female with pyoderma gangrenosum and IgA myeloma	Colchicine 0.5 mg/day: no benefit	No

**Table 2:** Alternatives to dapsone therapy for subcorneal pustular dermatosis reported in the last 25 years (1998–2023).

Successful treatment	Case	Patient	Successful treatment course
*Antibiotics*			
Doxycycline	Korbi et al. [23]	54-year-old female	Doxycycline 100 mg/day for 3 months, decreased to 50 mg/day for 3 months: remission after 4 weeks. No relapse or adverse effect at 13-month follow-up

*Corticosteroids (oral)*			
Betamethasone	Ceccarelli et al. [24]	92-year-old male with monoclonal IgG gammopathy	Betamethasone 3 mg once daily (tapered at week 2) and topical mometasone furoate which was replaced by methylprednisolone aceponate topical emulsion and emollients at week 2: improvement after 1 week with complete resolution after 5 months

Prednisolone	Ranieri et al. [25]	93-year-old female	Prednisolone 25 mg/day taper for 10 days: improvement, with relapse 2 weeks later. Resumption of steroids resulted in complete remission after 6 weeks
	Brown et al. [21]	78-year-old female with chronic lymphocytic leukemia	Prednisolone 20 mg/day: improvement within 7 days
	Lotery et al. [26]	29-year-old female with congenital cyanotic heart disease	Prednisolone, dapsone, and topical corticosteroids: improvement within an unknown duration of time

Cyclosporin	Karadoğan et al. [27]	50-year-old female	Cyclosporin 3 mg/kg/day and prednisone 1 mg/kg/day: gradual remission in 2 weeks
	Zachariae et al. [28]	29-year-old male	Cyclosporin 100–400 mg/day and prednisolone 35–100 mg/day: improvement within 2 days and no new lesions after 15 days

Intravenous immunoglobulin	Rasch et al. [29]	83-year-old male with combined lack of IgG/IgM and monoclonal IgA/kappa gammopathy	IVIG 0.2 g/kg: remission within a few days
	Kundak et al. [30]	5-year-old female with IgA elevation	IVIG 600 mg/kg: improvement within one week

*Monoclonal antibodies*			
Guselkumab	Teraki and Sugai [11]	72-year-old female with mild IgA elevation	Guselkumab 100 mg at baseline, one month, and then bimonthly: complete remission with no relapse at 12-month follow-up

*PDE4-inhibitors*			
Apremilast	Magdaleno-Tapial et al. [31]	65-year-old female	Apremilast 30 mg BID: significant improvement at 5 weeks

*Phototherapy and laser therapy*			
Psoralen UVA	Khachemoune and Blyumin [3]	28-year-old male	PUVA maintenance therapy, once every three weeks: significant improvement and control with maintenance therapy
	Bauwens et al. [32]	55-year-old male with monoclonal IgA gammopathy	PUVA three times a week and dapsone 50 mg/day: Improvement after 10 sessions, complete remission after 15 sessions

Narrowband UVB	Bordignon et al. [33]	28-year-old female	Narrowband UVB phototherapy three times a week and clobetasol ointment: complete remission after 42 treatment sessions with no relapse at 24-month follow-up
Excimer laser	Miura and Fujiwara [34]	83-year-old male	308-nm UVB excimer laser at maximal erythema dose (MED; 800 mJ·cm^−2^)/month: improvement after four sessions. After 24 sessions and 0.5 MED 12 sessions, sustained remission for 6 months with no treatment

*Purine biosynthesis inhibitors*			
Mizoribine	Kono et al. [35]	27-year-old female	Mizoribine 150 mg/day and 50 mg/day maintenance dose: dramatic improvement after 1 week. No relapses at 6-month follow-up

*Retinoids*			
Acitretin	Canpolat et al. [36]	55-year-old female with monoclonal IgA gammopathy	Acitretin 10–25 mg/day: improvement within 2 weeks with complete resolution at 4 months
	Young et al. [37]	33-year-old male with IgG MGUS	Acitretin 25–40 mg/day and clobetasol ointment BID: improvement at 4-week follow-up, complete resolution after increased dose (40 mg) for 4 weeks
	Ratnarathorn and Newman [15]	45-year-old female with nodal marginal zone lymphoma	Acitretin 50/25 mg (alternating dose) per day and rituximab (initiated to treat lymphoma): improvement noted after 1 year of rituximab and no relapses on maintenance acitretin
	Neely et al. [38]	58-year-old male with monoclonal IgA gammopathy	Acitretin 40 mg/day: complete response in 8 days, sustained at 15-month follow-up
	Yayli et al. [39]	10-year-old female	Acitretin 0.5 mg/kg/day: nearly complete resolution within 4 weeks. Reduced to every other day without relapses at 1-month follow-up
	Teixeira et al. [40]	78-year-old male	Acitretin 35 mg/day: improvement in 2 weeks

Etretinate	Hagino et al. [41]	71-year-old with IgG-Kappa multiple myeloma	Etretinate 20 mg/day for 10 days: remission with no recurrence at 7-month follow-up

*TNF inhibitors*			
Adalimumab	Guerin et al. [1]	69-year-old female with monoclonal IgA gammopathy	Adalimumab 40 mg every 2 weeks with dapsone 50 mg/day: complete remission after 1 month. Relapse occurred at 5 months but reducing interval to adalimumab 40 mg every week for 1 month caused clearance again which was sustained at 1-year follow-up
	Guerin et al. [1]	83-year-old female with monoclonal IgA gammopathy	Adalimumab 50 mg every 2 weeks: complete remission at 3 months. No recurrence after six months
	Chen et al. [42]	28-year-old female	Adalimumab 80 mg/week with acitretin 0.6 mg/kg/day and methylprednisolone 40 mg/day: improvement within 1 week
			Adalimumab 40 mg for one week, followed by 40 mg every two weeks, with acitretin and methylprednisolone tapers: remission at 6-week follow-up
Etanercept	Iobst and Ingraham [43]	27-year-old female with rheumatoid arthritis	Etanercept, unknown dose: resolution
	Bedi [10]	28-year-old female	Etanercept 25 mg biweekly with tacrolimus 0.1% ointment PRN: 80% improvement after 3 months and 100% improvement after 5 months
			Etanercept 50 mg biweekly w/o tacrolimus: complete remission after 3 months
	Berk et al. [16]	51-year-old male	Etanercept 50 mg twice weekly with acitretin 25 mg every other day: clinical regression after 1 month, maintained at 13-month follow-up
	Berk et al. [16]	61-year-old male	Etanercept 50 mg twice weekly with topical steroids PRN: improvement at 1-month follow-up, mild flare at 7 months, improvement again at 9-month follow-up with the same regimen

Infliximab	Kretschmer et al. [44]	29-year-old male	Infliximab 350 mg single dose: regression in a few days
			Maintenance therapy with infliximab started after 2 months: no relapses
	Voigtländer et al. [17]	79-year-old female	Infliximab 5 mg/kg with methylprednisolone 0.4 mg/kg and acitretin 0.4 mg/kg daily: improvement within 2 days after infliximab, with a few relapses when methylprednisolone was not part of therapy. With combination of all three, complete remission for 6 months
	Romagnuolo et al. [18]	80-year-old female	Infliximab, induction dose 5 mg/kg at weeks 0, 2, 6 and maintenance dose of 5 mg/kg every 8 weeks with dapsone 50 mg/day: improvement after one week and complete remission after one month
	Naretto et al. [19]	37-year-old female with systemic lupus erythematosus	Infliximab 5 mg/kg at weeks 0, 2, 6, 14, 22 and then every other month, plus prednisone and azathioprine: improvement within 24 hours with remission sustained at 6 months
	Bonifati et al. [14]	54-year-old female	Infliximab 5 mg/kg at weeks 0, 2, 6, 14, and 22 with methylprednisone and acitretin: improvement within 48 hours, however flare at week 12

*Topicals only*			
Topical steroids	Sauder and Glassman [45]	48-year-old female with rheumatoid arthritis taking adalimumab	Clobetasol propionate cream 0.05% BID: gradual improvement and complete resolution at 1 year
	Lade and Morey [46]	23-year-old female during pregnancy	Clobetasol propionate cream 0.05% BID: improvement within 7 days, mild flare after discontinuing, but resolved with resuming treatment for 2 weeks and had no relapses at 6-month follow-up
	Scheinfeld et al. [47]	61-year-old female with rheumatoid arthritis	Clobetasol propionate ointment: remission
	Lombart et al. [48]	36-year-old male with mycoplasma pneumoniae infection	Topical corticosteroids: complete remission in a few weeks with no recurrence at 18-months follow-up
	Barahimi et al. [49]	51-year-old male with Crohn's disease treated with ustekinumab	Topical steroids: controlled rash
	Bohelay et al. [50]	“Early 20s”-year-old male with mycoplasma pneumoniae infection	Topical steroids: complete remission in 1 week

Topical vitamin D derivatives	Hoshina et al. [51]	69-year-old female	Maxacalcitol: remission at 1 month, sustained at 4 months
	Kawaguchi et al. [52]	77-year-old male	Tacalcitol: improvement after 1 month with no relapse
			Strong corticosteroid ointment: improvement after two weeks but relapse after 3 months

Topical dapsone	Doolan et al. [53]	82-year-old female	Daily topical dapsone 7.5% gel: complete remission in 3 weeks

*Vitamin B derivatives*			
Riboflavin + nicotinamide	Yamaguchi et al. [54]	62-year-old male	Vitamin B2 riboflavin low dose and subsequent 1500 mg/day oral nicotinamide: gradual improvement with clearance at 2 months

*Xanthine derivatives*			
Pentoxifylline	Falcone et al. [55]	“20s”-year-old female	Pentoxifylline 400 mg TID: remission for 7 years, only one flare which was treated with prednisone

## Data Availability

The data used to support the findings of this study can be obtained from the corresponding author upon request.
